# Transcriptional profiling of cattle infected with *Trypanosoma congolense *highlights gene expression signatures underlying trypanotolerance and trypanosusceptibility

**DOI:** 10.1186/1471-2164-10-207

**Published:** 2009-05-01

**Authors:** Grace M O'Gorman, Stephen DE Park, Emmeline W Hill, Kieran G Meade, Paul M Coussens, Morris Agaba, Jan Naessens, Stephen J Kemp, David E MacHugh

**Affiliations:** 1 Animal Genomics Laboratory, UCD School of Agriculture, Food Science and Veterinary Medicine, UCD College of Life Sciences, University College Dublin, Belfield, Dublin 4, Ireland; 2 Comparative Immunology Group, School of Biochemistry and Immunology, Trinity College Dublin, Dublin 2, Ireland; 3 Department of Animal Science and Center for Animal Functional Genomics, Michigan State University, East Lansing, Michigan 48824, USA; 4 International Livestock Research Institute, Box 30709, Nairobi 00100, Kenya; 5 School of Biological Sciences, University of Liverpool, Liverpool L69 7ZD, UK; 6 UCD Conway Institute of Biomolecular and Biomedical Research, University College Dublin, Dublin 4, Ireland

## Abstract

**Background:**

African animal trypanosomiasis (AAT) caused by tsetse fly-transmitted protozoa of the genus *Trypanosoma *is a major constraint on livestock and agricultural production in Africa and is among the top ten global cattle diseases impacting on the poor. Here we show that a functional genomics approach can be used to identify temporal changes in host peripheral blood mononuclear cell (PBMC) gene expression due to disease progression. We also show that major gene expression differences exist between cattle from trypanotolerant and trypanosusceptible breeds. Using bovine long oligonucleotide microarrays and real time quantitative reverse transcription PCR (qRT-PCR) validation we analysed PBMC gene expression in naïve trypanotolerant and trypanosusceptible cattle experimentally challenged with *Trypanosoma congolense *across a 34-day infection time course.

**Results:**

Trypanotolerant N'Dama cattle displayed a rapid and distinct transcriptional response to infection, with a ten-fold higher number of genes differentially expressed at day 14 post-infection compared to trypanosusceptible Boran cattle. These analyses identified coordinated temporal gene expression changes for both breeds in response to trypanosome infection. In addition, a panel of genes were identified that showed pronounced differences in gene expression between the two breeds, which may underlie the phenomena of trypanotolerance and trypanosusceptibility. Gene ontology (GO) analysis demonstrate that the products of these genes may contribute to increased mitochondrial mRNA translational efficiency, a more pronounced B cell response, an elevated activation status and a heightened response to stress in trypanotolerant cattle.

**Conclusion:**

This study has revealed an extensive and diverse range of cellular processes that are altered temporally in response to trypanosome infection in African cattle. Results indicate that the trypanotolerant N'Dama cattle respond more rapidly and with a greater magnitude to infection compared to the trypanosusceptible Boran cattle. Specifically, a subset of the genes analyzed by real time qRT-PCR, which display significant breed differences, could collectively contribute to the trypanotolerance trait in N'Dama.

## Background

African animal trypanosomiasis (AAT), caused by the protozoan parasites *Trypanosoma congolense, T. vivax *and *T. brucei brucei*, is a wasting disease the affects cattle in much of central Africa. Transmission of the disease occurs though the saliva of infected tsetse flies (*Glossina *spp.). Characteristic waves of parasitaemia ensue with intermittent fever and the major clinical sign, anemia, develops [1,2]. Other symptoms include lymphoid enlargement, loss of condition and immunosuppression with reduced host resistance to secondary infections [1]. Human African trypanosomiasis (HAT), or sleeping sickness, caused by *T. brucei gambiense *and *T. brucei rhodesiense *infection is still a major public health problem in 36 African countries. It has been estimated that between 300,000 to 500,000 people are currently infected [3].

While other pathogens evade innate and adaptive responses in the plasma by hiding in a host cell, African trypanosomes are unique for being able to multiply and survive in the blood of their mammalian host [4]. In this regard, African trypanosomes have evolved an array of host evasion mechanisms, including the phenomenon of antigenic variation of the variable surface glycoprotein (VSG) to successfully inhabit the extracellular space, as in the case with *T. congolense*, in full view of the host immune system [5-7].

AAT is a major constraint to livestock production in Sub-Saharan African, where the disease is endemic. It occurs across an area of roughly seven million km^2^, puts approximately 60 million cattle at risk in 37 countries, and is estimated to cost livestock producers and consumers more than one billion US dollars annually [8]. Although the majority of cattle in Africa are susceptible to the disease, some West African *Bos taurus *cattle populations have evolved a level of tolerance to trypanosomiasis termed trypanotolerance [9]. These include the N'Dama breed, which offers the opportunity to study the mechanisms underlying trypanotolerance when contrasted with the response of a trypanosusceptible breed such as the East African Boran (*B. indicus*).

N'Dama cattle, although equally susceptible to the initial infection, survive and are productive in areas of tsetse challenge without the use of trypanocidal drugs [10]. This is achieved through a superior ability to control parasite proliferation, control anaemia and maintain body weight [4,11,12]. Research focused on the susceptible and tolerant host responses to trypanosome infection presents an opportunity to identify the poorly understood mechanisms underlying trypanotolerance.

A previous transcriptional profiling study with trypanosusceptible Boran used a cDNA microarray platform [13]. That study contributed to our understanding of the temporal transcriptional response of bovine peripheral blood mononuclear cells (PBMC) *in vivo *to a controlled trypanosome infection and identified time points with the greatest numbers of differentially expressed genes. Subsequently, a cytokine mRNA profiling study examined the immune response of both trypanosusceptible and trypanotolerant cattle PBMC to infection [14]. The cytokine profiling study reported that transcript levels for the *IL2, IL8 *and *IL1RN *genes were significantly downregulated across the time course in both breeds. Additionally, there were increases in transcripts for genes encoding proinflammatory mediators (*IFNG, IL1A, TNF*, and *IL12*) in N'Dama by 14 days dpi compared with pre-infection levels. And by peak parasitaemia, a type 2 helper T cell (T_H_2)-like cytokine environment was prevalent in the trypanosusceptible Boran with increases in transcripts for the *IL6 *and *IL10 *genes. Overall, the data suggested that the trypanotolerant N'Dama were more capable of responding very early in infection. Additionally, the trend of a greater magnitude of a T_H_2-like response in the Boran would not be expected to facilitate clearance of the trypanosomes and resolve infection.

In the present study, we have employed bovine long oligonucleotide (BLO) microarrays and real time quantitative reverse transcription PCR (qRT-PCR) to catalogue and analyze gene expression changes in PBMC from trypanotolerant and trypanosusceptible cattle following an experimental challenge with *T. congolense*. The results presented in this study significantly enhance our understanding of the global transcriptional response to trypanosome infection in mammalian PBMC. In addition, they also shed light on regulatory gene expression events that may underlie the phenomena of trypanotolerance and trypanosusceptibility in African cattle. This study reveals changes in the transcriptional profiles of genes involved in a diverse range of biological processes; the findings greatly expand on previous transcriptional profiling studies with trypanosusceptible Boran [13] and complements work describing cytokine responses of these animals to trypanosome infection [14].

## Results

### Overview of gene expression changes in N'Dama and Boran cattle

**Figure 1:**
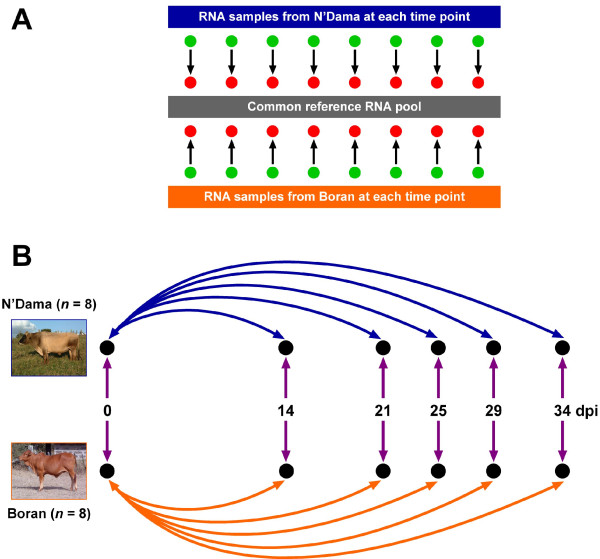
**Reference microarray hybridization schema and infection time course experimental design**. (A) The reference hybridization schema used for the microarray experiments. The common reference RNA pool was generated by combing equal quantities of all samples in the study, which included RNA from pre- and post-infection time points. Each arrow represents a single array hybridized with one experimental sample labelled with Alexa Fluor^® ^555 (green) and one common reference RNA sample labelled with Alexa Fluor^® ^647 (red). (B) The experimental design used for the trypanosome infection time course. The double-headed arrows show the gene expression contrasts that were catalogued across the infection time course for the trypanotolerant N'Dama breed (blue) and the trypanosusceptible Boran breed (orange). Gene expression contrasts between breeds at each time point are shown as double-headed purple arrows. Days post infection, dpi. The arrow colour coding for the gene expression contrasts corresponds to the colour coding of the graphs shown in Figs. 2 and 3.

**Figure 2:**
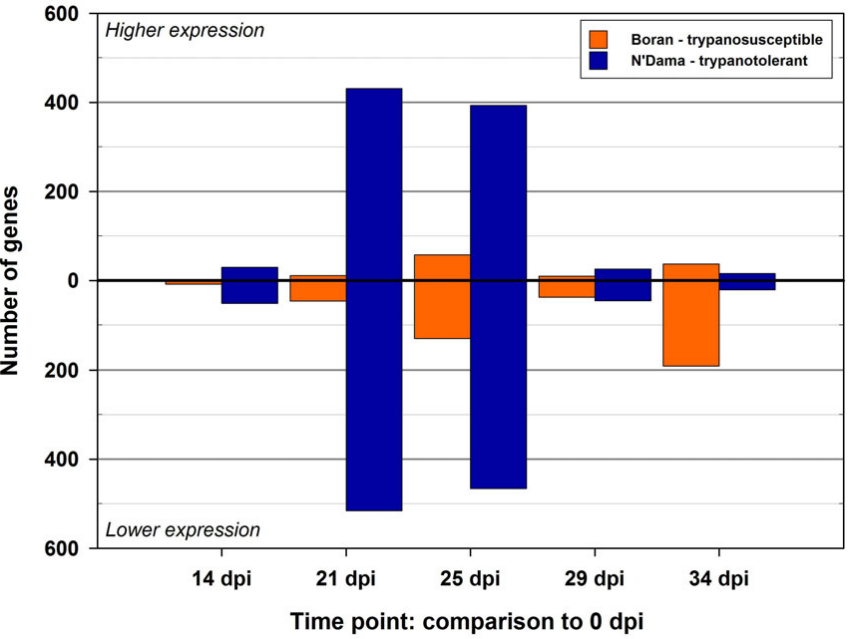
**Differentially expressed genes on the BLO microarray across the trypanosome infection time course**. Gene expression contrasts across the time course for each time point relative to 0 dpi are shown for the trypanosusceptible (Boran) and trypanotolerant (N'Dama) breeds after adjustment for multiple testing using the false discovery rate correction of Benjamini and Hochberg [52].

**Figure 3:**
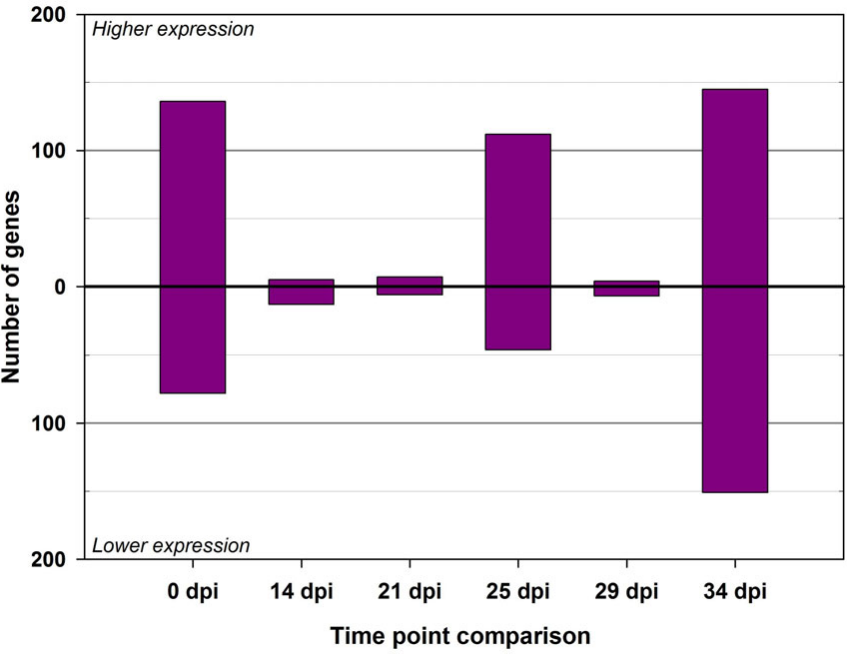
**Differentially expressed genes on the BLO microarray between breeds at each time point of the trypanosome infection time course**. Gene expression contrasts between the trypanosusceptible (Boran) and trypanotolerant (N'Dama) breeds breeds for each time point relative to 0 dpi are shown for the trypanotolerant (N'Dama) breed relative to the trypanosusceptible (Boran) breed after adjustment for multiple testing using the false discovery rate correction of Benjamini and Hochberg [52]. Direction of expression change is for the N'Dama breed relative to the Boran breed.

Fig. 1A shows the schema used for the microarray hybridizations between the individual animal PBMC RNA samples and a common reference RNA Pool. Fig. 1B shows the experimental design used for the trypanosome infection challenge experiments with eight Boran and eight N'Dama cattle; also shown are the gene expression contrasts across the time course for each breed, and the gene expression contrasts between breeds at each time point. The numbers of significantly differentially expressed genes detected either over the time course or between the breeds using BLO microarrays after adjustment for multiple testing using the false discovery rate correction (see Methods) are detailed in Table 1 and shown graphically in Figs. 2 and 3. The BLO microarray expression data generated was deposited in the NCBI Gene Expression Omnibus (GEO) repository [15] with experiment series accession [GEO: GSE14451].

**Table 1:** Differentially expressed genes detected over the trypanosome infection time course and between breeds.

**Comparison/contrast**	**Unexpressed**	**Unchanged**	**Increased expression**	**Decreased expression**	**% of** **microarray DE^b^**
Boran: 14 *vs *0 dpi	2,693	5,217	2	8	0.1
Boran: 21 *vs *0 dpi	2,511	5,352	11	46	0.7
Boran: 25 *vs *0 dpi	2,888	4,845	58	129	2.4
Boran: 29 *vs *0 dpi	2,220	5,653	10	37	0.6
Boran: 34 *vs *0 dpi	2,164	5,528	37	191	2.9
N'Dama: 14 *vs *0 dpi	2,287	5,552	30	51	1.0
N'Dama: 21 *vs *0 dpi	2,620	4,353	431	516	12.0
N'Dama: 25 *vs *0 dpi	2,551	4,510	393	466	10.8
N'Dama: 29 *vs *0 dpi	1,909	5,940	26	45	0.9
N'Dama: 34 *vs *0 dpi	2,871	5,012	16	21	0.5
N'Dama *vs *Boran: 0 dpi	2,628	5,078	136	78	2.7
N'Dama *vs *Boran: 14 dpi	2,360	5,542	5	13	0.2
N'Dama *vs *Boran: 21 dpi	2,509	5,398	7	6	0.2
N'Dama *vs *Boran: 25 dpi	2,754	5,008	112	46	2.0
N'Dama *vs *Boran: 29 dpi	1,805	6,104	4	7	0.1
N'Dama *vs *Boran: 34 dpi	2,189	5,435	145	151	3.7

^a ^DE: Differentially expressed

Expressed genes are defined as those having foreground ≥ background +2 SD in ≥ 50% of microarrays in any group of microarrays for a contrast.

Numbers of differentially expressed genes (*P *≤ 0.05) are based on a total of 7,920 genes and are FDR-adjusted.

For the between breed contrasts, increased expression indicates higher in N'Dama compared to Boran and decreased expression indicates higher in Boran compared to N'Dama.

The greatest numbers of differentially expressed genes (*P *≤ 0.05) were evident in PBMC from infected N'Dama at 21 dpi and 25 dpi (0 *vs *21 dpi, *n *= 947 and 0 *vs *25 dpi, *n *= 859). These changes represented 12% and 11% of all gene features on the BLO microarray at 21 and 25 dpi respectively. Overall, the total number of significant differentially expressed genes at each time point indicated temporal differences in the response of trypanotolerant N'Dama and trypanosusceptible Boran cattle to trypanosome infection (for example, 0 *vs *14 dpi: *n *= 8 genes in Boran and *n *= 81 genes in N'Dama). This translated to a 10-fold difference in the magnitude of response in N'Dama relative to Boran at the earliest post-infection time point of 14 dpi (0.1% in Boran compared to 1.0% in N'Dama) compared to pre-infection. Generally, throughout the time course, a trend emerged where more genes showed decreased expression within each group post-infection relative to pre-infection. Although, for some contrasts (when much higher numbers of differentially expressed genes were involved in the estimation) comparable numbers of genes increased and decreased in expression [0 *vs *21 dpi in N'Dama, *n *= 431 genes increased (45.5%) and *n *= 516 genes decreased (54.5%) and 0 *vs *25 dpi in N'Dama, *n *= 393 genes increased (45.8%) and *n *= 466 genes decreased (54.2%)]. The greatest magnitude of differences in responses between breeds, over the time course, was apparent at 34 dpi with a total of 296 genes detected as differentially expressed, including 145 showing increased expression in N'Dama relative to Boran and 151 increased in Boran relative to N'Dama representing 3.7% of genes on the BLO microarray. The range in fold changes detected using the BLO microarray across the time course varied from 4.82-fold decreased expression to 3.68-fold increased expression.

### Analysis of Over-Represented Functional Categories

The most significantly over-represented gene ontology (GO) categories, classified according to biological process (*P *≤ 0.01) are presented for each breed over time, and between breeds in Table 2. Over-representation analysis of gene ontology showed that by 14 dpi, the Boran had increased GO categories representation in a broad range of processes, which were not directly or obviously involved in the immune response, including sodium ion transport, sphingolipid catabolism and neurotransmitter uptake. At the same time point, for GO category classified genes in the N'Dama, an increase in representation of genes involved in RNA and mRNA metabolic processes was apparent. Later in the time course, a predominance of immune-related processes was evident in the GO classification. At 21 dpi, the oxygen and reaction oxygen species metabolism and T cell proliferation processes were over-represented in the Boran group; while at the same time point in the N'Dama group, a wide range of processes were detected as over-represented including regulation of cytokine production, endocytosis, regulation of chemotaxis and leukocyte activation. At the final time point of 34 dpi an increase in genes involved in translation, signal transduction and defence response was observed in the Boran group relative to pre-infection. Concomitantly, differentially expressed genes in N'Dama showed a higher proportion of genes involved in defence responses, signalling and immunoglobulin mediated immune responses. In relation to breed differences, a relatively large number of biological processes were detected as significantly over-represented before infection and that number was only surpassed at 34 dpi when a larger number of diverse over-represented processes were detected including, erythrocyte differentiation, translation, response to biotic stimulus, defence response, amino acid biosynthesis and RNA export from the nucleus.

**Table 2:** Biological process gene ontology (GO) categories significantly over-represented in N'Dama and Boran over the time course (P ≤ 0.01).

	**Comparison/contrast**		
	
**Time point**	**Boran** **(time point *vs *0 dpi)**	**N'Dama (time point *vs *0 dpi)**	**Boran *vs *N'Dama**
**0 dpi**	Not applicable	Not applicable	Eating behaviour Regulation of RNA metabolic process Regulation of cytokine biosynthetic process Negative regulation of inflammatory response Regulation of multicellular organismal process Regulation of cytokine production Chromatin assembly
**14 dpi**	Sodium ion transport Heparan sulfate proteoglycan metabolic process Lactation Neurotransmitter uptake Sphingolipid catabolic process	Regulation of RNA metabolic process mRNA metabolic process	Transport
**21 dpi**	Cell-cell signalling Adult locomotory behaviour Telomere maintenance Oxygen and reactive oxygen species metabolic process T cell proliferation	Fat cell differentiation Regulation of cytokine production Regulation of cytokine biosynthetic process Endocytosis Anatomical structure morphogenesis Regulation of multicellular organismal process Negative regulation of biosynthetic process Synaptic transmission, dopaminergic Cell motility Embryonic development Regulation of chemotaxis Leukocyte activation Protein polymerization	Defence response
**25 dpi**	Translation Activation of JNK activity DNA methylation Androgen receptor signalling pathway Actin polymerization and/or depolymerization Sphingolipid catabolic process Response to hydrogen peroxide	Anatomical structure morphogenesis Regulation of cytokine biosynthetic process Endocytosis Regulation of cytokine production	Translation Defence response Sphingolipid catabolic process Response to biotic stimulus
**29 dpi**	Regulation of protein amino acid phosphorylation	Cytoskeleton organization and biogenesis	Response to biotic stimulus
**34 dpi**	Translation Regulation of protein amino acid phosphorylation Ras protein signal transduction Regulation of tyrosine phosphorylation of Stat3 protein Defence response Cell-cell signalling Neurotransmitter uptake Oxygen and reactive oxygen species metabolic process	Defence response Response to biotic stimulus Oxygen and reactive oxygen species metabolic process Immunoglobulin mediated immune response Transmembrane receptor protein tyrosine kinase signalling pathway Antimicrobial humoral response (sensu Vertebrata)	Urea cycle Arginine metabolic process Erythrocyte differentiation Translation Response to biotic stimulus Defence response Chondroitin sulfate biosynthetic process Anterior/posterior axis specification Histone ubiquitination Amino acid biosynthetic process Xenobiotic metabolic process RNA export from nucleus

### Real time qRT-PCR profiles of candidate genes in N'Dama and Boran

**Figure 4:**
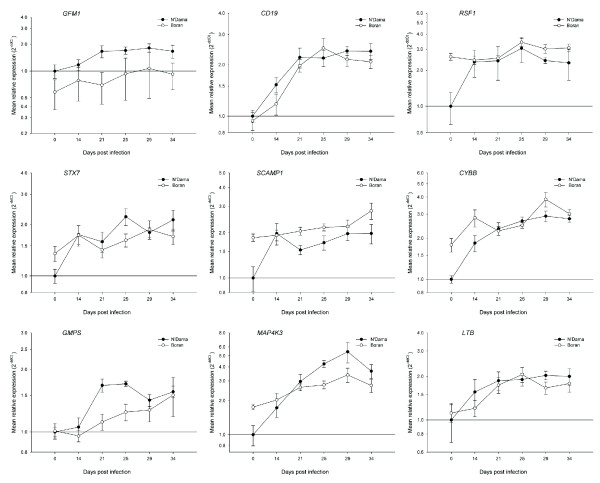
**Real time qRT-PCR results for nine selected genes from trypanotolerant N'Dama and trypanosusceptible Boran cattle across the trypanosome infection time course**. Data analysis was carried out using the standard 2^-ΔΔCt ^method [55]. All *C*_t _values were corrected using the housekeeping gene *PPIA *and the N'Dama group at time point 0 was used as the calibrator. Each point on the graph (common log scale) represents the mean fold change in gene expression relative to pre-infection levels ± SEM. Genes shown are: *GFM1*, *CD19*, *RSF1*, *STX7*, *SCAMP1*, *CYBB*, *GMPS*, *MAP4K3 *and *LTB*.

**Figure 5:**
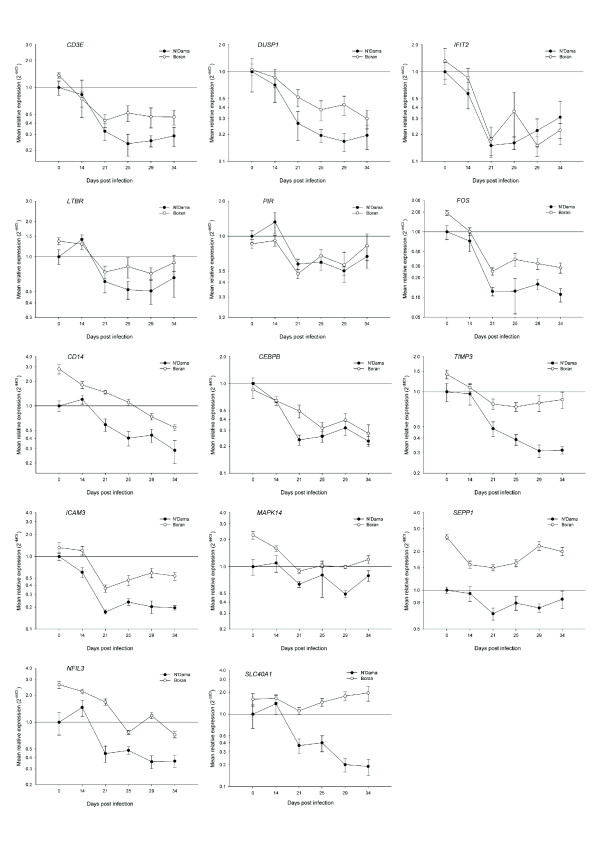
**Real time qRT-PCR results for 14 selected genes from trypanotolerant N'Dama and trypanosusceptible Boran cattle across the trypanosome infection time course**. Data analysis was carried out using the standard 2^-ΔΔCt ^method [55]. All *C*_t _values were corrected using the housekeeping gene *PPIA *and the N'Dama group at time point 0 was used as the calibrator. Each point on the graph (common log scale) represents the mean fold change in gene expression relative to pre-infection levels ± SEM. Genes shown are: *CD3E*, *DUSP1*, *IFIT2*, *LTBR*, *PIR*, *FOS*, *CD14*, *CEBPB*, *TIMP3*, *ICAM3*, *MAPK14*, *SEPP1*, *NFIL3*, and *SLC40A1*.

**Figure 6:**
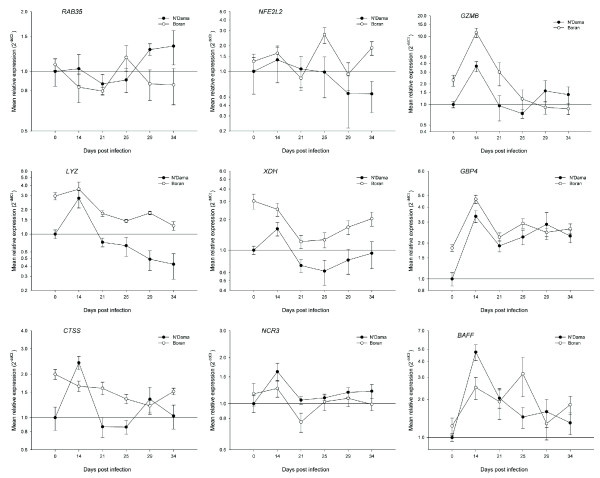
**Real time qRT-PCR results for nine selected genes from trypanotolerant N'Dama and trypanosusceptible Boran cattle across the trypanosome infection time course**. Data analysis was carried out using the standard 2^-ΔΔCt ^method [55]. All *C*_t _values were corrected using the housekeeping gene *PPIA *and the N'Dama group at time point 0 was used as the calibrator. Each point on the graph (common log scale) represents the mean fold change in gene expression relative to pre-infection levels ± SEM. Genes shown are: *RAB35*, *NFE2L2*, *GZMB*, *LYZ*, *XDH*, *GBP4*, *CTSS*, *NCR3 *and *BAFF*.

In light of the wide range of biological processes over-represented in the ontology analysis in response to trypanosome infection, a diverse set of genes was subsequently selected from the output of the microarray data analysis for real time qRT-PCR validation. A list of 32 genes (see Table 3 for major functions) involved in many different physiological processes including: regulation of transcription, regulation of protein biosynthesis, immune response, intracellular protein transport, protein phosphorylation, dephosphorylation, biosynthetic pathways and response to stress were selected for microarray data validation and further investigation of the differentially activated molecular mechanisms. The graphs shown in Figs. 4, 5 and 6, which represent the mean fold changes, show the changing mRNA profiles for each breed over the entire time course. Overall, similar trends in mRNA expression emerged for subsets of profiles including, the most frequently observed trends of a general increase, or a general decrease in expression over time in one or both breeds. Another pattern of expression detected was an early increase (at 14 or 21 dpi) in expression followed by either a decrease or no further change in one or both breeds.

**Table 3:** Biological process gene ontologies for genes validated by real time qRT-PCR.

**Gene** **Symbol**	**Gene Name**	**Gene Ontology: Biological Process**
*BAFF*	B cell activating factor, TNFSF13B	B cell co-stimulation
CD3E	T-cell receptor CD3 epsilon chain	T cell receptor signalling pathway
*CD14*	CD14 antigen	Inflammatory response
*CD19*	CD19 antigen	B cell receptor signalling pathway
*CEBPB*	CCAAT/enhancer binding protein beta	Inflammatory response
*CTSS*	Cathepsin S	Proteolysis
*CYBB*	Cytochrome b-245, beta polypeptide	Inflammatory response
*DUSP1*	Dual specificity phosphatase 1	Intracellular signalling cascade
*FOS*	Murine FBJ osteosarcoma viral (v-fos) oncogene homolog	Regulation of transcription, DNA-dependent
*GBP4*	Guanylate binding protein 4-like	Immune response
*GFM1*	Elongation factor G1	Translation elongation
*GMPS*	Guanine monphosphate synthetase	Purine base biosynthetic process
*GZMB*	Granzyme B precursor	Induction of apoptosis by granzyme
*ICAM3*	Intercellular Adhesion Molecule 3	Biological process unknown
*IFIT2*	Interferon-induced protein with tetratricopeptide repeats 2	Biological process unknown
*LTB*	Lymphotoxin-beta isoform a	Immune response
*LTBR*	Lymphotoxin beta receptor	Positive regulation of NF-KappaB cascade
*LYZ*	Lysozyme	Defense response to bacterium
*MAP4K3*	Mitogen-activated protein kinase kinase kinase kinase 3	Protein kinase cascade, response to stress
*MAPK14*	Mitogen-activated protein kinase 14	Protein kinase cascade, response to stress
*NCR3*	Natural cytotoxiciy triggering receptor 3	Inflammatory response
*NFE2L2*	Nuclear factor (erythroid-derived 2)-like 2	Regulation of transcription
*NFIL3*	Nuclear factor, interleukin 3 regulated	Immune response
*PIR*	Pirin	Transcription from RNA polymerase II promoter
*RAB35*	Member RAS oncogene family	Protein localization, endosome transport
*RSF1*	Remodelling and spacing factor 1	Chromatin remodelling
*SCAMP1*	Secretory carrier-associated membrane protein 1	Post-Golgi vesicle-mediated transport
*SEPP1*	Selenoprotein P, plasma, 1	Response to oxidative stress
*SLC40A1*	Solute carrier family 40 (iron-regulated transporter), member 1	Cellular iron ion homeostasis
*STX7*	Syntaxin-7	Post-Golgi vesicle-mediated transport
*TIMP3*	Tissue inhibitor of metalloproteinase 3	Transmembrane receptor protein tyrosine kinase signalling pathway
*XDH*	Xanthene dehydrogenase	Purine base metabolic process

The first nine genes from the panel of 32 are shown in Fig. 4 and generally increase in expression for one or both groups of cattle and include the expression profiles for *GFM1*, *CD19*, *RSF1*, *STX7*, *SCAMP1*, *CYBB*, *GMPS*, *MAP4K3 *and *LTB*. The mRNA for the G elongation factor mitochondrial 1 protein (GFM1), one of three factors required by the elongation stage of the mitochondrial translation system, significantly increased in expression in PBMC from the N'Dama cattle over time and was consistently higher in N'Dama compared to Boran at all time points. The expression of the *GFM1 *gene failed to increase significantly in PBMC from the Boran cattle over the time course; PBMC from N'Dama, on the other hand, displayed highly significant increases at 25 and 29 dpi in particular compared to pre-infection levels (1.7-fold, *P *= 0.0095 and 1.8-fold, *P *= 0.0085 respectively). The difference in expression detected at 21 dpi, 2.4-fold higher in N'Dama compared to Boran (*P *= 0.0002), was one of the most significant breed differences detected in this study.

Significant, parallel increases in the mRNA expression level for the CD19 molecule, a membrane co-receptor found on all B cells were observed in N'Dama and Boran post-infection. The highly significant two- to three-fold increases were particularly evident after 14 dpi, when parasites were first apparent in the blood. In particular, the *CD19 *gene was highly significantly increased in expression in PBMC from N'Dama (*P *= 0.0000) and Boran (*P *= 0.0001) at 29 dpi relative to pre-infection. Endogenous mRNA levels of the chromatin remodelling and spacing factor 1 gene (*RSF1*) were significantly different between PBMC from N'Dama and Boran before infection (2.6-fold higher in Boran, *P *= 0.0109). However, following experimental infection, the profiles of *RSF1 *expression in both breeds showed very similar overall levels between breeds as N'Dama significantly increased in expression to a maximal level of 3.1-fold, *P *= 0.0126, at 25 dpi relative to pre-infection while expression in Boran remained relatively stable throughout.

The *STX7 *gene – encoding a protein involved in post-Golgi vesicle-mediated trafficking of proteins from the plasma membrane to endosomes and lysosomes – is another example of a gene with significantly increased expression in PBMC from N'Dama over time while remaining relatively stable in PBMC from Boran cattle. At 25 dpi, in particular, when maximum levels of *STX7 *mRNA were observed in N'Dama (2.2-fold, *P *= 0.0013) compared to pre-infection, N'Dama had 1.4-fold higher levels of *STX7 *mRNA relative to Boran (*P *= 0.0484). In addition to *STX7*, the secretory carrier membrane protein 1 gene (*SCAMP1*) is also categorized under the gene ontology biological process termed 'post-Golgi vesicle-mediated protein transport.' Before infection, the Boran group displayed a significant 1.9-fold higher level of *SCAMP1 *mRNA compared to N'Dama (*P *= 0.0109); however, expression levels remained reasonably constant after infection with no significant changes detected. Conversely, N'Dama, although starting with almost two-fold lower levels of the *SCAMP1 *transcript had significantly increased expression of *SCAMP1 *by 1.5 to 2.0-fold over the time course. At 29 dpi, in particular, a highly significant two-fold increase in expression of the *SCAMP1 *gene was detected in PBMC from the N'Dama group (*P *= 0.0031) relative to pre-infection levels.

The cytochrome b-245, beta polypeptide gene (*CYBB*) encodes a gp-91 phox (phagocyte oxidase) protein, which is a critical component of the microbicidal oxidase system of phagocytes. The *CYBB *gene was observed to increase in mRNA expression in PBMC from both breeds relative to pre-infection over the time course. However, the increase in expression in the N'Dama group was more uniform and sustained over time (although not significantly higher than the Boran at any time point measured) resulting in highly significant increases in *CYBB *expression from 21 dpi onwards (2.3-fold, *P *= 0.0007 at 21 dpi; 2.7-fold, *P *= 0.0000 at 25 dpi, 2.9-fold, *P *= 0.0002 at 29 dpi and 2.8-fold, *P *= 0.0000 at 34 dpi relative to pre-infection).

The guanine monophosphate synthetase gene (*GMPS*), which encodes a protein involved in the *de novo *synthesis of guanine nucleotides (essential for DNA and RNA synthesis and elevated in rapidly growing cells) was highly significantly increased in expression in PBMC from N'Dama after 14 dpi post-infection until the end of the time course relative to pre-infection levels. During this time, the expression levels in Boran were never significantly different to pre-infection levels. N'Dama showed an increase in expression that was particularly evident at 21 and 25 dpi, (1.7-fold, *P *= 0.0005 and 1.7-fold, *P *= 0.0000) respectively, which resulted in significant breed differences at those times (1.5-fold, *P *= 0.0022 at 21 dpi and 1.4-fold, *P *= 0.0052 at 25 dpi, higher in N'Dama). Therefore, although the absolute mean mRNA levels were similar before infection and at 34 dpi for both breeds, during the first wave of parasitaemia, N'Dama expressed significantly higher levels of the *GMPS *transcript.

Mitogen-activated protein kinase kinase kinase kinase 3 (MAP4K3), also known as germinal centre kinase like kinase (GLK), is a member of the Ser/Thr protein kinase family and is thought to function in response to stress, specifically activating the Jun N-terminal kinase (JNK) signalling pathway. Despite an almost significant 1.8-fold higher level of *MAP4K3 *mRNA in the Boran group relative to N'Dama before infection (*P *= 0.0617); The N'Dama group had significantly higher expression levels of *MAP4K3 *at 25 dpi (1.5-fold, *P *= 0.0000) 29 dpi (1.6-fold, *P *= 0.0166) and 34 dpi (1.3-fold, *P *= 0.0058) relative to the Boran. This was the result of highly significant increases in *MAP4K3 *expression in N'Dama that were evident at the time points after 14 dpi (3.0-fold, *P *= 0.0021 at 21 dpi; 4.2-fold, *P *= 0.0000 at 25 dpi; 5.4-fold, *P *= 0.0047 at 29 dpi and 3.7-fold, *P *= 0.0005 at 34 dpi). These changes in N'Dama were coupled with moderate and less significant increases in the Boran group (1.5-fold, *P *= 0.0293 at 21 dpi and 1.6-fold, *P *= 0.0416 at 25 dpi) relative to pre-infection levels.

The lymphotoxin beta gene (*LTB*) involved in the inflammatory response and the normal development of lymphoid tissues, showed increasing levels of expression in the N'Dama and Boran groups that were significant after 14 dpi. Increases were comparable for both breeds with maximal levels in N'Dama at 29 dpi (2.0-fold, *P *= 0.0089) and 25 dpi in Boran (1.8-fold, *P *= 0.0126) compared to pre-infection. A marginally higher response was observed for the N'Dama: a 1.2-fold higher level of *LTB *expression relative to Boran at 29 dpi (*P *= 0.0190).

Fourteen genes (*CD3E*, *DUSP1*, *IFIT2*, *LTBR*, *PIR*, *FOS*, *CD14*, *CEBPB*, *TIMP3*, *ICAM3*, *MAPK14*, *SEPP1*, *NFIL3*, and *SLC40A1*) had profiles of expression that generally decreased over time in PBMC from one or both breeds after infection (Fig. 5). A subset of these genes (*CD3E*, *DUSP1*, *IFIT2*, *LTBR*, *PIR *and *FOS*) behaved similarly, displaying coordinated patterns of expression in PBMC from both the N'Dama and Boran groups with no significant breed differences in gene expression after trypanosome infection. Decreased expression was particularly marked for these genes from 21 dpi.

The CD3e molecule, epsilon (CD3-TCR complex) gene (*CD3E*) encodes a cell differentiation antigen, which is part of the TCR-CD3 complex of T-lymphocytes and is involved in the positive regulation of T cell proliferation through generation of intracellular signal when antigen is bound to the TCR. Highly significant decreases in *CD3E *mRNA were detected in PBMC at 21 dpi (3.1-fold, *P *= 0.0079 and 3.2-fold, *P *= 0.0001 in N'Dama and Boran respectively) relative to pre-infection levels, which was sustained throughout the time course. Similarly, the dual specificity phosphatase 1 gene (*DUSP1*), which is a key regulator of the immune response through its role in the dephosphorylation and inactivation of MAP kinases, had significantly decreased expression from 25 dpi relative to pre-infection in PBMC from the N'Dama and Boran groups. The interferon-induced protein with tetratricopeptide repeats 2 gene (*IFIT2*) displayed an mRNA expression profile that showed a sharp decrease in PBMC at 21 dpi compared to pre-infection that was comparable for both breeds.

The lymphotoxin beta receptor (TNFR superfamily, member 3) gene (*LTBR*) encodes a receptor for the heterotrimeric lymphotoxin membrane form (a complex containing the LTA and LTB proteins), which is involved in the development of lymphoid tissue, the immune response and apoptosis. The expression of *LTBR *in PBMC from both animal groups generally decreased after 21 dpi; interestingly, however, a modest increase (1.4-fold, *P *= 0.0510) was detected at 14 dpi relative to pre-infection in the N'Dama group. Fluctuations in PBMC mRNA expression were observed for the pirin (iron-binding nuclear protein) gene (*PIR*), which encodes a transcriptional cofactor that interacts with the protein product of the nuclear factor I/C (CCAAT-binding transcription factor) gene (*NFIC*). However, there was a clear tight co-ordinate response in PBMC expression between N'Dama and Boran over the whole time course. The v-fos FBJ murine osteosarcoma viral oncogene homolog gene (*FOS*) displayed an expression profile that showed highly significant and substantial decreases – again, in a coordinated fashion – between the N'Dama and Boran groups, which were particularly apparent from 21 dpi onwards relative to pre-infection levels.

Five of the subset of genes shown in Fig. 5 that generally decreased in expression across the time course (*CD14*, *CEBPB*, *TIMP3*, *ICAM3 *and *MAPK14*) were similarly regulated in the N'Dama and Boran groups and were comparable to the genes described above; however, the magnitude of response varied between the breeds and resulted in significant differences between the N'Dama and Boran groups at one or more time points. The CD14 molecule gene (*CD14*), an LPS- and apoptotic cell-binding molecule that is preferentially expressed on monocytes and macrophages had 2.5-fold (*P *= 0.0031) and 2.7-fold (*P *= 0.0439) lower levels of expression at 21 and 25 dpi respectively in PBMC from the N'Dama group relative to the Boran animals.

The PBMC mRNA expression profiles of the CCAAT/enhancer binding protein (C/EBP), beta gene (*CEBPB*) in the N'Dama and Boran groups tracked each other across the time course with the exception of 21 dpi, where the Boran had 2.1-fold (*P *= 0.0071) higher levels of *CEBPB *mRNA relative to the N'Dama. The PBMC gene expression profiles of the TIMP metallopeptidase inhibitor 3 gene (*TIMP3*) diverged from 21 dpi, after which the Boran appeared to stabilize and the N'Dama continued to decrease in expression of *TIMP3 *mRNA. This breed divergence in *TIMP3 *expression subsequently resulted in higher levels of *TIMP3 *mRNA expression in the Boran relative to the N'Dama at 25 dpi (1.9-fold, *P *= 0.0101), 29 dpi (2.6-fold, *P *= 0.0329) and 34 dpi (2.7-fold, *P *= 0.0299).

The mRNA expression profiles for the intracellular adhesion molecule 3 gene (*ICAM3*) were consistently lower in PBMC from the N'Dama compared to the Boran animals at 14 dpi (2.0-fold, *P *= 0.0306), 21 dpi (2.1-fold, *P *= 0.0096), 29 dpi (2.9-fold, *P *= 0.0124) and 34 dpi (2.7-fold, *P *= 0.0087). Over the entire time course, modest changes in expression occurred for the mitogen-activated protein kinase 14 gene (*MAPK14*). Despite this, at 29 dpi a significantly greater mRNA abundance of *MAPK14 *was observed in PBMC from Boran relative to N'Dama (2.0-fold, *P *= 0.0008).

Three genes that generally decreased in expression over the time course displayed PBMC expression profiles that varied significantly between the N'Dama and Boran groups (*SEPP1*, *NFIL3 *and *SLC40A1*). Although selenoprotein P, plasma 1 gene (*SEPP1*) mRNA expression decreased in N'Dama and Boran over time; at almost all time points examined, Boran had significantly higher levels of *SEPP1 *mRNA relative to N'Dama. Similarly, expression of a transcriptional activator, the nuclear factor, interleukin 3 regulated gene (*NFIL3*) generally decreased in both breeds over time; however, at 21 dpi the Boran displayed a highly significant 3.8-fold (*P *= 0.0066) increase of *NFIL3 *mRNA relative to the N'Dama. The solute carrier family 40 (iron-regulated transporter), member 1 gene (*SLC40A1*), which plays an essential role in iron ion homeostasis produced PBMC mRNA expression profiles across the time course that were among the most divergent in terms of differences in response between breeds. Expression levels of *SCL40A1 *mRNA did not significantly change in Boran over time, although there was tendency to increased expression; however, expression levels in the N'Dama were markedly depressed at 29 dpi (5.0-fold, *P *= 0.0417) and 34 dpi (5.3-fold, *P *= 0.0398) relative to pre-infection levels. These changes resulted in significantly higher levels of *SLC40A1 *mRNA in Boran relative to N'Dama at 21 dpi (3.0-fold, *P *= 0.0059), 25 dpi (3.6-fold, *P *= 0.0098), 29 dpi (8.8-fold, *P *= 0.0043) and 34 dpi (10.3-fold, *P *= 0.0311).

The mRNA expression profiles of two genes across the time course (*RAB35 *and *NFE2L2*) showed a pattern of early coordinate expression followed by later divergence in PBMC from the two groups of animals (Fig. 6). The RAB35, member RAS oncogene family gene (*RAB35*) encodes a member of the Rab family, which are major regulators of intracellular protein transport. Minor fluctuations in expression are observed before 25 dpi; however, by 29 dpi N'Dama displayed higher mRNA levels of *RAB35 *mRNA relative to Boran (1.5-fold, *P *= 0.0377). Conversely, the expression levels of the nuclear factor (erythroid-derived 2)-like 2 gene (*NFE2L2*) were reduced after 25 dpi in N'Dama (although not significantly) while they fluctuated in Boran to a large extent over the time course. The result at 34 dpi was a 3.5-fold higher level of *NFE2L2 *mRNA in the Boran relative to the N'Dama (*P *= 0.0164).

The final series of PBMC expression profiles consists of seven genes also shown in Fig. 6 (*GZMB*, *LYZ*, *XDH*, *GBP4*, *CTSS*, *NCR3 *and *BAFF*) with mRNA levels that followed a trend of increasing early with a subsequent decrease or no further increase in one or both breeds. The first five of these genes (*GZMB*, *LYZ*, *XDH *and *GBP4*) have comparable expression profiles for N'Dama and Boran and therefore have no significant breed differences after infection; the last three (*CTSS*, *NCR3 *and *BAFF*), on the other hand, have diverging profiles and significant differences between N'Dama and Boran at either 14 or 21 dpi. The granzyme B (granzyme 2, cytotoxic T-lymphocyte-associated serine esterase 1) gene (*GZMB*) encodes a protease necessary for target cell lysis in cell-mediated immune responses. The *GZMB *gene exhibited a significant peak in mRNA expression at 14 dpi in both breeds (3.7-fold, *P *= 0.0031 in N'Dama and 5.0-fold, *P *= 0.0291 in Boran) relative to pre-infection values. Expression levels of the lysozyme (renal amyloidosis) gene (*LYZ*) did not change significantly in PBMC from the Boran over time, although they did tend to decrease over time. In contrast to this, the N'Dama showed an initial 2.8-fold increase (*P *= 0.0286) in *LYZ *mRNA abundance at 14 dpi relative to pre-infection that was followed by later decreases in expression. A single, modest but significant, increase in xanthine dehydrogenase gene (*XDH*) expression was detected at 14 dpi in N'Dama relative to pre-infection levels (1.6-fold, *P *= 0.0382). Guanylate binding protein 4 gene (*GBP4*) PBMC expression profiles for the N'Dama and Boran were synchronized after infection, with an early highly significant peak in expression at 14 dpi (3.3-fold, *P *= 0.0002 in N'Dama and 2.6-fold, *P *= 0.0029 in Boran) followed by a reduction in expression in both breeds.

The three last gene expression profiles (*CTSS, NCR3 *and *BAFF*) exhibit trends of early increases in expression in one or both breeds with significant differences between N'Dama and Boran at 14 or 21 dpi. The cathepsin S gene (*CTSS*) encodes a thiol protease that is responsible for the removal of the invariant chain from MHC class II molecules, thereby functioning in MHC class II-associated chain processing and peptide loading in the immune response. The mRNA expression profiles of *CTSS *expression in PBMC from N'Dama and Boran over the time course were strikingly different. There was no increase in *CTSS *mRNA abundance in Boran post-infection; Conversely, a highly significant increase in *CTSS *gene expression was observed for the N'Dama group at 14 dpi relative to pre-infection (2.4-fold, *P *= 0.0004). These fluctuations resulted in a 1.5-fold higher level of *CTSS *mRNA in N'Dama relative to Boran at 14 dpi (*P *= 0.0258). The natural cytoxicity triggering receptor 3 gene (*NCR3*) that may contribute to the increased efficiency of activated NK cells to lyse cells in the inflammatory response, showed a significant increase in gene expression in the N'Dama group at 14 dpi (1.6-fold, *P *= 0.0258) relative to pre-infection. Additionally, although *NCR3 *mRNA expression was reduced in N'Dama at 21 dpi, there was a significant 1.4-fold higher level of *NCR3 *mRNA (*P *= 0.0190) in N'Dama relative to Boran at this time. The tumour necrosis factor (ligand) superfamily, member 13 b gene (*TNFSF13B*) that encodes a potent B cell activating cytokine (*BAFF*), which plays an important role in the proliferation and differentiation of B cells, is abundantly expressed in peripheral blood leukocytes. In this study, *TNFSF13B *mRNA expression was highly significantly elevated in PBMC from N'Dama at 14 dpi (4.7-fold, *P *= 0.0008) and significantly increased at 21 dpi (2.0-fold, *P *= 0.0169) relative to pre-infection levels. Despite moderate fluctuations in *TNFSF13B *expression over time in the Boran, no significant differences were detected relative to pre-infection levels. Finally, at 14 dpi PBMC from the N'Dama had 1.9-fold higher mRNA levels of *TNFSF13B *relative to the Boran (*P *= 0.0129).

## Discussion

This study describes large-scale transcriptional profiling of PBMC from naïve trypanosusceptible Boran and trypanotolerant N'Dama using bovine long oligonucleotide (BLO) microarrays following experimental *T. congolense *infection. A number of differentially expressed genes were selected from the results of the microarray analysis for real time qRT-PCR validation. This study builds on previous work that encompassed immunospecific microarray analyses of Boran experimentally infected with trypanosomes [13] and cytokine mRNA profiling with immune cell subpopulation analyses [14]. When the physiological parameters of parasitaemia and packed cell volume are taken into account, the findings described here can be placed in a broader and more meaningful setting. Within this context, the opportunity therefore exists to examine not only how the host responds to infection over time, but also to consider possible mechanisms that might contribute to trypanotolerance as represented by the superior response of N'Dama cattle to trypanosome infection.

### Phenotypic responses across the infection time course

As previously described, the cattle in this study exhibited typical phenotypic responses as measured by parasitaemia and packed red blood cell volume (PCV) [14]. The kinetics of parasitaemia showed the first peak of parasitaemia occurring between 15–22 dpi. Rates of decline in PCV were similar for N'Dama and Boran up to 22 dpi but thereafter the kinetics of anaemia diverged and the decline in PCV in Boran was more pronounced than that for N'Dama, such that the Boran displayed significantly lower PCV measures compared to the N'Dama at 26 and 32 dpi. Also, broadly speaking there were increases in the proportion of B cells (following an initial decrease at 14 dpi) and reductions in the proportions of various T cell subpopulations (CD4^+^, CD8^+ ^and δγ T cells) over the course of infection.

### Gene expression contrasts and gene expression changes for the two breeds

The numbers of differentially expressed genes for each contrast (between time points within breeds or between breeds at the same time point) were tabulated and are presented in Table 1. The most striking feature from these figures is the larger number of genes that were classified as differentially expressed in N'Dama at 21 and 25 dpi (at peak parasitaemia) relative to pre-infection compared to any other contrast. The numbers of differentially expressed genes represented approximately 12.0% and 10.8% of all gene features on the BLO microarray at 21 and 25 dpi respectively. These data suggest that there are chronological differences in the transcriptional response of N'Dama and Boran to trypanosome infection. This is also apparent at the early time point of 14 dpi, where an approximate 10-fold higher magnitude of response was observed in N'Dama relative to Boran compared to pre-infection. It is therefore likely that the relatively rapid transcriptional response observed in N'Dama after infection may contribute to a more favourable outcome; perhaps reflecting early innate events while the adaptive immune response is established.

In general, comparisons of the differential expression profiles showed more genes decreased in expression in response to experimental infection, than were induced. For the many contrasts examined across the time course, it is important to note, that more genes were decreased than increased in expression (although for some comparisons the numbers were comparable). This may seem counterintuitive for an immune response that is being mounted towards a blood-borne parasite; however, the effect of managing an appropriate immune response in an effort to limit immunopathology (by downregulation of inflammatory response) [16] and the possibility of host-pathogen interactions (leading to immunosuppression) [17] could give rise to a downregulation of host gene expression. Additionally, the preponderance of genes decreased in expression may reflect a decrease in the proportions of cell types expressing these transcripts, highlighting the importance of the between breed contrasts. With this in mind, the greatest magnitude of breed difference over the time course was apparent at 34 dpi. It appears that the numbers of genes differentially expressed between N'Dama and Boran initially decreases, so that the breeds become more similar at 14 dpi than they were before infection. Considered together, these results indicate that the differences observed prior to infection may simply reflect the different genetic backgrounds of the two populations. However, when challenged with the same pathogen, where neither breed is resistant to the initial infection, their transcriptional profiles (at least at 14 dpi) are relatively similar. Subsequently, at 34 dpi, the N'Dama and Boran cattle become more divergent than they were before infection. Differences in the breed profiles at this time reflect the diverging response to infection between the trypanotolerant and trypanotolerant cattle, which was also evident from their PCV scores.

### Gene ontology over-representation analysis: involvement of diverse cellular processes

A useful method to understand the predominating responses is GO classification of differentially expressed genes. The response to trypanosome infection in the two breeds was also examined using gene ontology (GO) over-representation analysis of the BLO microarray gene expression data (Table 2). The over-represented ontology categories are generated from differentially expressed genes and may therefore contain genes with increased and decreased expression within each category, thereby representing overall increased activity for a particular biological process. A number of general observations can be made based on the over-representation results, which only included the most significant findings (*P *≤ 0.01). Firstly, although only represented by relatively few genes, the early response in Boran at 14 dpi relative to pre-infection was characterized by a diverse range of ontology categories including sodium ion transport, sphingolipid catabolism and neurotransmitter uptake. At the same time the response in N'Dama was characterized entirely by RNA and mRNA metabolic processes, which may reflect a heightened activation status in the PBMC of N'Dama at 14 dpi. At the height of parasitaemia, further immune-related processes were represented in addition to a range of physiological processes. By 21 dpi compared to pre-infection in Boran, oxygen and reaction oxygen species metabolism, T cell proliferation and cell-cell signalling processes were over-represented. These processes could reflect a changing host environment where parasitaemia is now high and the susceptible host is struggling to mount a successful immune response. Concurrently, the response in N'Dama is characterized by increased expression for genes involved in a range of biological processes including regulation of cytokine production and chemotaxis, endocytosis, cell motility and leukocyte activation – all of which are expected to be beneficial for the host.

As the infection time course advanced, the ontology categories increasingly included those related to signalling and the defence response. By 34 dpi, cellular processes involved in translation, regulation of amino acid phosphorylation, ras protein signal transduction, oxygen and reactive oxygen species metabolic processes and the defence response were affected in Boran relative to pre-infection. Biological processes affected in the N'Dama group at 34 dpi also included oxygen and reactive oxygen species metabolic processes and defence response; however, additional ontology categories included response to biotic stimulus, immunoglobulin mediated immune responses and antimicrobial humoral response. It is noteworthy that breed differences with respect to the number of differentially expressed genes were high before infection and maximal at 34 dpi. This resulted in a variety of biological processes that were affected or had genes over-represented differently between N'Dama and Boran. This included genes involved in arginine metabolic processes, erythrocyte differentiation, translation, response to biotic stimulus, defence response, amino acid biosynthesis and RNA export from the nucleus. The diversity of ontology categories over-represented at 34 dpi between trypanotolerant N'Dama and trypanosusceptible Boran, would support the hypothesis that the mechanisms underlying the phenomenon of trypanotolerance may be varied and multifaceted [11,18]. In light of the observation that a range of cellular processes underlie the phenomenon of trypanotolerance, a diverse panel of genes were chosen as candidates for real time qRT-PCR validation (see Table 3).

### Evidence for heightened B cell responses in trypanotolerant N'Dama cattle

Evidence of a rise in B cell responses including antigen processing and presentation was evident in the transcriptional profiles of a number of genes. These include genes involved in processes such as post-Golgi vesicle-mediated protein transport (*STX7 *and *SCAMP1*), regulation of intracellular protein trafficking (*RAB35*), MHC Class II-associated chain processing and peptide loading (*CTSS*), and B cell activating factor (*BAFF*) in addition to the B cell co-receptor (*CD19*). These processes are all involved in mounting a successful B cell response to infection.

Post-Golgi vesicle-mediated processes are represented by both *STX7 *and *SCAMP1 *in this study. Syntaxin 7 (encoded by the *STX7 *gene), is a member of a family of transmembrane proteins that have been implicated as vesicle receptors involved in vesicle docking and fusion and STX7 has been proposed to have a role in vesicle trafficking between the Golgi complex and lysosomes and has been shown to be associated with the early endosome [19,20]. Secretory carrier membrane proteins (SCAMPs) 1–4 are ubiquitously expressed and major components of the eukaryotic cell surface recycling system that shuttles between the plasma membrane, endosomes and the trans-Golgi complex [21] and have been shown to participate in endocytosis [22]. *STX7 *and *SCAMP1 *transcript levels, which generally increased over the time course, were only significantly increased in expression in N'Dama relative to pre-infection and at 25 dpi N'Dama had 1.4-fold higher levels of *STX7 *mRNA relative to Boran.

*RAB35 *encodes a protein that is a member of the RAB family, which is localized to the plasma membrane and endocytic compartments, is involved in the regulation of the endocytic pathway [23]. After the first wave of parasitaemia an increase in *RAB35 *mRNA expression was apparent in N'Dama, which was significantly but modestly higher relative to Boran at 29 dpi.

The cathepsin S gene (*CTSS*) encodes a lysosomal cysteine protease that is responsible for the removal/processing of the invariant chain from MHC class II molecules, thereby functioning in MHC class II-associated chain processing, peptide loading and maturation. The profiles of *CTSS *expression in N'Dama and Boran over time were remarkably different; there was no increase in *CTSS *expression in Boran post-infection and in general the trend of expression was towards decreased expression. Conversely, a highly significant increase in *CTSS *expression for N'Dama at 14 dpi relative to pre-infection was observed. The result of these different trends was higher *CTSS *mRNA abundance in N'Dama relative to Boran at the early time point of 14 dpi, when parasites were just appearing in the bloodstream.

The transcript for B cell-activating factor gene (*BAFF*) encodes a potent B cell activating cytokine that has important roles in B-cell homeostasis including B-cell growth, survival, proliferation and differentiation [24,25]. In this study, *BAFF *mRNA expression was highly significantly elevated in N'Dama at 14 dpi and 21 dpi relative to pre-infection levels and despite moderate fluctuations in *BAFF *expression over time in Boran, no significant differences were detected relative to pre-infection levels. Significantly at 14 dpi, N'Dama had higher mRNA levels of *BAFF *relative to Boran.

The parallel, highly significant, increase in *CD19 *expression (a membrane co-receptor found on all B cells) in N'Dama and Boran might indicate that *CD19 *plays a key role in the response to trypanosome infection and that role is equally employed by both tolerant and susceptible cattle. The timing of the response has an appropriate association with the function of CD19 in the host immune response to infection, where significant increases in *CD19 *expression were detected after parasites were first apparent in the bloodstream and highly significant increases were detected at peak parasitaemia. The innate immune response of microbial antigen recognition by complement is coupled to the activation of B cells though the membrane protein complex CD19/CD21 [26]. This occurs through CD21 binding of the C3d fragment of activated C3 that is covalently attached to targets of complement activation (in this case, trypanosome antigens) and subsequently CD19 co-stimulates or enhances signalling through the B cell receptor [27]. Early increases in *CD19 *expression, after parasites and parasite antigens are accessible, is clearly an important and fundamental response to infection mounted by both breeds of cattle, allowing the integration of innate and adaptive immune recognition at the B cell to subsequently promote an appropriate adaptive immune response. Cumulatively, these findings indicate increased activity in B cell responses, specifically B cell co-receptor enhanced signalling, post-Golgi vesicle-mediated protein transport, MHC class II maturation, which are complemented by increased levels of the B cell activating factor. These responses appear to be more pronounced in the N'Dama than in the Boran cattle after infection and the overall result would feasibly result in a more efficient or appropriate B cell response in trypanotolerant cattle.

### Additional Immune responses

A number of additional genes that play important roles in the immune response to infection were also increased in expression in response to trypanosome infection (*GZMB*, *LYZ*, *CYBB *and *NCR3*). Granzyme B, the product of the *GZMB *gene, is a serine protease that is an important mediator of target-cell apoptosis (granule exocytosis pathway) by cells such as natural killer cells (NK) and cytotoxic CD8^+ ^T cells [28]. The early increase in expression of *GZMB *mRNA may indicate a role for this serine protease in response to trypanosome infection, important in both breeds when parasites are first apparent in the bloodstream.

Lysozyme (encoded by *LYZ*) and cytochrome b-245 (encoded by *CYBB*) are both involved in the killing of microbes when phagocytosis has occurred [29]. Lysozyme, is involved in the lysosomal-dependent killing mechanism where the phagosome containing ingested microbes fuses with lysosomes to form a phagolysosome [29]. Cytochrome b-245, on the other hand, is involved in the lysosome-independent (fusion of phagosome with lysosome is not required) killing mechanism, such that oxygen radicals are generated inside the phagocytic vacuole that kill the microbe. The initial increase in expression of the *LYZ *gene in PBMC from N'Dama may be beneficial in the killing of trypanosomes ingested by the mononuclear phagocytic system in the phagolysosome of these cells. A highly significant and sustained increase in the expression of *CYBB *mRNA expression was detected in N'Dama after infection over the entire time course. Overall, however, the increase in *CYBB *expression suggests an important role for this molecule in the response to trypanosome infection in tolerant and susceptible cattle.

The natural cytoxicity triggering receptor 3 gene (*NCR3*) encodes the NKp30 protein and is expressed on resting and activated natural killer cells [30]. NKp30 is one of a group of triggering receptors responsible for positive NK cell stimulation and the process of natural cytotoxicity [31]. Notably, a recent study reported an association with a promoter polymorphism in the *NCR3 *gene and mild malaria attacks and a haplotype that contained the polymorphism was significantly associated with increased risk of mild malaria [32]. In the present study a significant increase in *NCR3 *expression was detected in N'Dama at 14 dpi, when parasites appeared in the bloodstream, relative to pre-infection. This expression pattern could possibly contribute to a superior NK cell function at this critical time in trypanotolerant cattle.

### Nucleic acid synthesis and the stress response

Transcripts for the *GMPS *and *MAP4K3 *genes showed significant breed differences post-infection where the trypanotolerant N'Dama had higher levels of expression. The guanine monophosphate synthetase gene (*GMPS*) encodes an enzyme involved in the *de novo *synthesis of guanine nucleotides (essential for DNA and RNA synthesis) and is elevated in rapidly growing cells [33]. At 21 and 25 dpi, significantly higher levels of *GMPS *mRNA were detected in N'Dama. The higher levels of *GMPS *during this time could be an indication of the activation status of cells and might suggest that N'Dama had, at this critical time, an elevated activation status where cells were growing more rapidly compared to Boran.

The mitogen-activated protein kinase kinase kinase kinase 3 gene (*MAP4K3*), encodes a member of the Ser/Thr protein kinase family that functions in response to stress, specifically activating the Jun N-terminal kinase (JNK) signalling pathway and is thought to have a particular role in germinal centre B cells [34-36]. Despite the higher endogenous levels in Boran before infection, N'Dama had significantly higher expression levels of *MAP4K3 *mRNA at 25, 29 and 34 dpi relative to Boran perhaps indicating a heightened response to stress in trypanotolerant cattle.

### Regulation of iron transport and homeostasis

Anaemia is a significant clinical sign of advancing trypanosome infection; therefore, genes involved in the regulation of iron transport and homeostasis represent interesting candidates to study. One such gene is the solute carrier family 40 (iron-regulated transporter), member 1 gene (*SLC40A1*), which plays an essential role in iron ion homeostasis [37,38].

Expression levels of *SLC40A1 *remained relatively stable in the trypanosusceptible Boran, while at the same time they were significantly depressed in N'Dama at 29 and 34 dpi relative to pre-infection. The result of this was significantly higher levels of *SLC40A1 *mRNA observed in Boran relative to N'Dama at 21 dpi, 25 dpi, 29 dpi and 34 dpi. The product of the *SLC40A1 *gene, ferroportin 1, is a main iron export protein. A decrease in ferroportin 1 expression, as seen in N'Dama, may result in reduced iron export and an increase in intracellular iron levels. High levels of iron, however, are known to be toxic and the action of reduced iron export on trypanosome growth is unknown, therefore the functional consequences of the divergent expression pattern of *SLC40A1 *in N'Dama and Boran remains to be fully elucidated.

### Other cellular processes

One of the genes examined using real time qRT-PCR (*GFM1*) displayed significantly higher expression levels, particularly at 21 dpi, in the trypanotolerant N'Dama compared to Boran. The protein product encoded by the nuclear gene *GFM1 *specifically promotes the GTP-dependent translocation of the nascent protein chain from the A-site to the P- site of the ribosome; therefore, GFM1 plays a crucial role in maintaining normal mitochondrial function through translation of mitochondrial encoded genes, including those of the enzyme complexes that perform oxidative phosphorylation [39,40]. Elongation factors involved in mitochondrial protein biosynthesis could conceivably contribute to the overall rate of translation, resulting in greater mitochondrial mRNA translational efficiency with potential benefit for trypanotolerant cattle.

## Conclusion

In summary, the transcriptional profiles of a diverse range of genes in PBMC are altered in response to trypanosome infection in both tolerant and susceptible cattle. Some of these genes appear to be regulated in a similar manner irrespective of breed and are not considered to be involved in trypanotolerance, while others exhibit significant breed differences in expression following infection. Indeed temporal differences in the magnitude of the response have suggested that N'Dama respond more rapidly to infection and by 34 dpi the response to infection is qualitatively different between the breeds as suggested by the over-representation analysis of GO categories. The combined effect of a number of these factors (involved in B cell activation pathways, innate immune responses, cytotoxic responses and other pathways) may result in the superior ability of N'Dama to control infection.

The results presented here demonstrate that genes regulating a diverse range of physiological processes contribute to the general host response to AAT and to the specific responses of trypanotolerant N'Dama cattle. Further resolution of the significance of these processes could be attained in future experiments on individual cell populations. Future work on the findings presented could include data mining studies, specifically of conserved regulatory sequences in the promoter regions of differentially expressed genes using tools such as MatInspector and databases such as MatBase [41,42]. Furthermore, the relationship between the expression of differentially expressed genes that code for secreted proteins and clinical measures of disease could also be fully explored, possibly offering additional insights into the mechanisms of trypanotolerance and trypanosusceptibility.

## Methods

### Animals and experimental infection

The work presented here is based on an experimental infection that was previously described [14]. Briefly, eight Boran (*B. indicus*, trypanosusceptible) and eight N'Dama (*B. taurus*, trypanotolerant) female cattle, aged between 19–28 months, were raised together at the International Livestock Research Institute (ILRI) farm at Kapiti Plains Estate, Kenya in an area free from trypanosomiasis. The cattle were each experimentally infected with the *T. congolense *clone IL1180 [43,44], which was delivered via the bites of eight infected tsetse flies [*G. morsitans morsitans*] [45,46]. Infections were carried at the ILRI laboratories at Nairobi, Kenya and all procedures were approved by the Institutional Animal Use and Care (IAUC) Committee of ILRI in concordance with the Animals (Scientific Procedures) Act (United Kingdom) 1986.

### Blood collection, PBMC isolation and RNA extraction and purification

Peripheral blood (200 ml) was collected before infection and at 14, 21, 25, 29 and 34 dpi in heparanized syringes. Fig. 1B shows the experimental design used for the trypanosome infection time course. These time points were chosen to coincide with the first wave of parasitaemia. PBMC were isolated using Percoll™ gradients (GE Healthcare, ) and previously described methods [47]. The isolated PBMC were washed with sterile PBS and stored immediately at -80°C in Tri-reagent (Molecular Research Center, Inc, ) for further processing. Total RNA was extracted with chloroform and precipitated with isopropanol. Each sample was further DNase-treated with RQ1 RNase-free DNase (Promega Corp., ) and purified using the RNeasy^® ^mini kit (Qiagen, ). RNA quality and quantity was subsequently assessed using the 18S/28S ratio and RNA integrity number (RIN) on an Agilent Bioanalyzer with the RNA 6000 Nano LabChip^® ^kit (Agilent Technologies, Inc., ).

### The bovine long oligo (BLO) microarray

The NCBI GEO platform accession for the BLO microarray is GPL3810. The BLO microarray platform has 17,328 spot features representing approximately 7,920 predicted bovine mRNA's spotted in duplicate supplemented with positive (*GAPDH*, *RPL19*, *RPLP2*, *28S RNA*, *60S RNA*, *PPIA*, *ACTB*, *PGK1*, *B2M *and *GUSB*), negative and artificial control sequences spotted multiple times. The oligonucleotides (70-mers) were designed and synthesized commercially by Operon Biotechnologies (Operon Biotechnologies, Inc., ). The oligonucleotides were designed to the The Institute for Genomic Research (TIGR) tentative consensus (TC) sequences (release October 5^th ^2004).

### cDNA conversion, labelling and hybridization to bovine long oligonucleotide microarrays

Labelled cDNA was generated using the SuperScript™ Plus Direct cDNA labelling System (Invitrogen Corp., ) according to the manufacturers' instructions. Briefly, 8 μg of sample RNA was labelled with Alexa Fluor^® ^555 and 10 μg of common reference RNA (CRR) was labelled with Alexa Fluor^® ^647. A common reference RNA pool was generated by combining equal quantities of all samples in the study, which included RNA from pre- and post-infection time points. Fig. 1A illustrates the common reference design employed. Labelled samples were purified using the purification module included with the SuperScript™ Plus Direct cDNA labelling System, combined and supplemented with approximately 60–70 μl SlideHyb Glass Hybridization Buffer #3 (Ambion Inc., ) to a final probe volume of 100 μl.

Probes were hybridized to bovine long oligonucleotide (BLO) microarrays, using an automated HS400 hybridization station (Tecan UK Ltd., ) with the following optimized protocol; WASH: 75°C, Runs 1, Wash 10 s, Soak 20 s; PROBE INJECTION: 85°C; Denaturation: 95°C, 2 mins; HYBRIDISATION: 42°C – 4 hrs; 35°C – 4 hrs; 30°C – 4 hrs; agitation frequency medium, WASH: 37°C, Runs 2, Wash 10 s, Soak 20 s; 25°C, Runs 2, Wash 15 s, Soak 30 s; 25°C, Runs 2, Wash 20 s, Soak 40 s and SLIDE DRYING: 25°C for 2 mins.

### Data collection, normalization, quality control and statistical analysis

Microarrays were scanned using a GenePix^® ^4000 B scanner (Molecular Devices Corp., ). The microarray hybridizations represented 5–8 animals of each breed at all of the time points (0, 14, 21, 25, 29 and 34 dpi) where the CRR served as the reference sample in each case (for a total of 88 slides). Spot features where foreground was less than background plus two standard deviations were flagged as lowly expressed. The linear models for microarray data (LIMMA) package in Bioconductor was used to identify differential gene expression [48,49]. Background correction was performed according to the robust multichip average (RMA) method [50]. An intensity-based normalization method was employed: within microarray print-tip dependent locally weighted scatterplot smoothing (LOWESS) normalization, followed by between microarray quantile normalization [51].

For each gene, moderated *t*-tests were used to compare between breeds or time points to determine differentially expressed genes. Probability values were corrected for multiple testing using the false discovery rate (FDR) correction of Benjamini and Hochberg [52]. For any given contrast, either between breeds or time points, genes flagged as lowly expressed in more than half of the microarrays in all sample groups under comparison were filtered out. FDR-adjusted, significantly differentially expressed genes (*P *≤ 0.05) were subjected to gene ontology (GO) over-representation analysis. For each GO term, the proportion of differentially expressed genes sharing each GO term was compared to the proportion for the whole microarray to identify terms showing significantly different proportions. This was accomplished using the GOHyperGAll function in Bioconductor which in turn uses the GOstats package [48,53]. GO terms with over-representation probability values of *P *≤ 0.01, ≥ 2 associated differentially expressed genes, ≤ 1000 associated genes in all, and that lack an immediate parent or immediate descendent terms with lower *P *values and the conditions above were retained.

### cDNA synthesis, real time quantitative RT-PCR (qRT-PCR) and data analysis

For the purposes of qRT-PCR, 4 μg of total RNA from each sample was reverse transcribed into cDNA with oligo-dT primers using a SuperScript™ III first strand synthesis SuperMix kit according to the manufacturer's instructions (Invitrogen Corp., ). The converted cDNA was quantified using a NanoDrop^® ^ND-1000 spectrophotometer (NanoDrop Technologies, Inc., ), diluted to 20 ng/μl working stocks and stored at -20°C for subsequent analyses. Primers for real time qRT-PCR were designed using the Vector NTI Advance™ software package (Invitrogen Corp., ) and commercially synthesized (Invitrogen Corp., ). Details for these primer sets are provided in Table 4. Each reaction was carried out in a total volume of 25 μl with 2 μl of cDNA (20 ng/μl), 12.5 μl 2 × PCR master mix (BioGene Ltd, ), 1.25 μl SYBR Green I (a 1/40,000 dilution with DMSO, BioGene Ltd, ) and 9.25 μl primer/H_2_O. Optimal primer concentrations were determined by titrating 100, 300 and 900 nM final concentrations and disassociation curves were examined for the presence of a single product. Real time qRT-PCR was performed using an MX3000P^® ^quantitative PCR system (Stratagene Corp., ) with the following cycling parameters: 95°C for 10 mins (PCR hot start) followed by 45 cycles of 95°C for 15 s and 60°C for 1 min, followed by amplicon dissociation (95°C for 1 min, 55°C for 30 s, increasing 0.5°/cycle for 81 cycles). The most stable reference gene for this challenge experiment was previously determined from a panel of putative reference genes and based on geNorm analysis [54], the peptidylprolyl isomerase A gene (*PPIA*) alone was used as the reference gene in this study [14]. The  method (where *C*_*T *_is cycle threshold) was used to determine mean fold changes in gene expression between breeds at a particular time point and between time points for each breed [55]. Student's *t*-test was used to identify significant differences in gene expression between breeds and time points. Because of differences in expression before infection, graphs presented here (Figs. 4, 5 and 6) have been scaled to take account of breed differences before infection, such that Boran fold changes are relative to the N'Dama fold changes that have been scaled to a pre-infection value of 1.0.

**Table 4:** Bovine oligonucleotide primers used for real time qRT-PCR.

**Gene symbol**	**Gene name**	**Primer sequence (5'-3')^a^**	**Amplicon size**	**Primer concentration**
*PPIA*	Peptidylprolyl isomerase A	F-CATACAGGTCCTGGCATCTTGTCC R-CACGTGCTTGCCATCCAACC	108 bp	300 nM
*BAFF*	B cell activating factor, TNFSF13B	F- GGAGATGAACTCCAACTGGCAA R- GTGAGCAGGTCACAGAAGCTTC	102 bp	100 nM
*CD3E*	T-cell receptor CD3 epsilon chain	F- CTGGTGTATTACTGGAGCAAGAGC R- GGGCTCATAGTCTGGATTGG	132 bp	300 nM
*CD14*	CD14 antigen	F- GGTCTCTCAATTTGTCGTTCGC R- GATTTCCGTCCAGAGTCAGG	153 bp	900 nM
*CD19*	CD19 antigen	F- CTTGGGTCCCAATCCTATGAGG R- CATGATTAGCACCAGGCTGG	91 bp	300 nM
*CEBPB*	CCAAT/enhancer binding protein beta	F- TGCAACGCCTGGTGGTCTGG R- CAGCCAAGCAGTCCGCCTCG	111 bp	300 nM
*CTSS*	Cathepsin S	F- AGGAAAGCTGGTGTCTCTGAGTGC R- TGTCATGAAGCCGCCATTGC	91 bp	300 nM
*CYBB*	Cytochrome b-245, beta polypeptide	F- TGGGAACCCTCCTATGACTTGG R- TGACCACCTTGGTGATGACC	116 bp	300 nM
*DUSP1*	Dual specificity phosphatase 1	F- CCTTGATCAACGTCTCTGCG R- TCCACCAGCATTCTTGATGG	152 bp	100 nM
*FOS*	Murine FBJ osteosarcoma viral (v-fos) oncogene homolog	F- CAATAACTACTGTGTTCCTGGC R- TCAGACCACCTCAACAATGC	186 bp	300 nM
*GBP4*	Guanylate binding protein 4-like	F- CCCATGCAAAGACCAAGACC R- ACTGATCCACTGTTGATGGC	103 bp	300 nM
*GFM1*	Elongation factor G1	F- GTTCCTTTGTGACAACCTGG R- CTACTTCTCTGCTCTCTGCG	159 bp	300 nM
*GMPS*	Guanine monphosphate synthetase	F- AAGTCTGCACTCACTGAATGC R- TAAGAGATTCCCAGTCAGGC	125 bp	300 nM
*GZMB*	Granzyme B precursor	F- ACCTACGCTGACACACTACAGG R- ACATTGTCACACACGAGAGG	173 bp	300 nM
*ICAM3*	Intercellular adhesion molecule 3	F- GATGATGGACGTTCAAGGTCG R- AAGACGTACACTGAGGCTGC	105 bp	300 nM
*IFIT2*	Interferon-induced protein with tetratricopeptide repeats 2	F- CCAGTATGTCAAAGTCCTCC R- ATAGCAAGGTCCAAGTCACC	165 bp	100 nM
*LTB*	Lymphotoxin-beta isoform a	F- CTACAATAAACCCTCCACGG R- CAGACAGGACATCTCCATCC	117 bp	900 nM
*LTBR*	Lymphotoxin beta receptor	F- CCAGGTCAGGAACATGTGTG R- GCCTCTGGTCTCATTTCTGC	176 bp	900 nM
*LYZ*	Lysozyme	F- CTCAGTCAACATGAAGGCTCTCG R- ACACAACCAGTTTGCCAGGC	154 bp	900 nM
*MAP4K3*	Mitogen-activated protein kinase kinase kinase kinase 3	F- TTTGAGTCACAGAATAGCGG R- TGAACTGATTCCCTCTATGG	155 bp	900 nM
*MAPK14*	Mitogen-activated protein kinase 14	F- GCTGTCGACCTGCTGGAGAAGATGC R- TCGTCGTCAGGATCGTGGTACTGG	110 bp	300 nM
*NCR3*	Natural cytotoxiciy triggering receptor 3	F- TAGAGACGCCAAGAGTGGAAGG R- AACCTCTCGCGAAGATAAGC	158 bp	300 nM
*NFE2L2*	Nuclear factor (erythroid-derived 2)-like 2	F- CAACCTTTGTCGTCATCACAGG R- GGGAATGGGATATGGAGAGC	194 bp	900 nM
*NFIL3*	Nuclear factor, interleukin 3 regulated	F- CCAGAAGTGAATTCCTCTGCCTTGC R- TTAGACGTCATATCAACAGGCGAGG	134 bp	100 nM
*PIR*	Pirin	F- TTAAATTGGACCAAGGAGCG R- ACAAAGTGGCTTCTCTCAGG	196 bp	900 nM
*RAB35*	Member RAS oncogene family	F- GAACGATCAGGGTCTTCTGC R- GATGTGATTGTCCAGTCAGG	173 bp	900 nM
*RSF1*	Remodelling and spacing factor 1	F- TGCATTCCAAATCTGTGAGG R- GCAACAATACGGACTTGAAC	157 bp	900 nM
*SCAMP1*	Secretory carrier-associated membrane protein 1	F- TACAGACCACTTTACGGAGC R- GAAATCCAACCACAGTTACC	140 bp	300 nM
*SEPP1*	Selenoprotein P, plasma, 1	F- CCCTACTTCAGGTCTTCATCACC R- TGCATCTCTTTCGTCAGAGC	143 bp	300 nM
*SLC40A1*	Solute carrier family 40 (iron-regulated transporter), member 1	F- ATAATACAGTGAAGGCACGAGG R- CAAGACAGAGATCCTGAAGG	130 bp	300 nM
*STX7*	Syntaxin-7	F- ATCTGGTGGTTTTCCTGAGG R- TCTTCATCCTGCAACTGAGC	93 bp	300 nM
*TIMP3*	Tissue inhibitor of metalloproteinase 3	F- ATCTCCCAGACTCTCCTTCC R- ACAGTTGGCTTTTCAGCAGC	91 bp	300 nM
*XDH*	Xanthene dehydrogenase	F- CTCCGTACAGACATTGTCATGG R- GGAATAGTGTAGCTCCTCCAGG	123 bp	300 nM

^a ^F: Forward primer, R: Reverse primer.

## Authors' contributions

GMOG, EWH, MA, JN, SJK and DEM conceived and designed the experiments. GMOG, EWH, KM and MA performed the experiments. GMOG, SDEP and DEM analyzed the data. PMC contributed reagents/materials/analysis tools. GMOG, SDEP, JN and DEM wrote the paper. All authors read and approved the final manuscript.

## References

[B1] Darji A, Lucas R, Magez S, Torreele E, Palacios J, Sileghem M, Bajyana Songa E, Hamers R, De Baetselier P (1992). Mechanisms underlying trypanosome-elicited immunosuppression. Ann Soc Belg Med Trop.

[B2] Murray M, Dexter TM (1988). Anaemia in bovine African trypanosomiasis. A review. Acta Trop.

[B3] Cattand P, Jannin J, Lucas P (2001). Sleeping sickness surveillance: an essential step towards elimination. Trop Med Int Health.

[B4] Naessens J (2006). Bovine trypanotolerance: A natural ability to prevent severe anaemia and haemophagocytic syndrome?. Int J Parasitol.

[B5] Borst P, Cross GA (1982). Molecular basis for trypanosome antigenic variation. Cell.

[B6] Donelson JE (2003). Antigenic variation and the African trypanosome genome. Acta Trop.

[B7] Pays E (2005). Regulation of antigen gene expression in *Trypanosoma brucei*. Trends Parasitol.

[B8] Kristjanson PM, Swallow BM, Rowlands GJ, Kruska RL, de Leeuw PN (1999). Measuring the costs of African trypanosomosis, the potential benefits of control and returns to research. Agric Syst.

[B9] Murray M, Morrison WI, Whitelaw DD (1982). Host susceptibility to African trypanosomiasis: trypanotolerance. Adv Parasitol.

[B10] Murray M, Trail JC, D'Ieteren GD (1990). Trypanotolerance in cattle and prospects for the control of trypanosomiasis by selective breeding. Rev Sci Tech.

[B11] Naessens J, Teale AJ, Sileghem M (2002). Identification of mechanisms of natural resistance to African trypanosomiasis in cattle. Vet Immunol Immunopathol.

[B12] Paling RW, Moloo SK, Scott JR, McOdimba FA, Logan-Henfrey LL, Murray M, Williams DJ (1991). Susceptibility of N'Dama and Boran cattle to tsetse-transmitted primary and rechallenge infections with a homologous serodeme of *Trypanosoma congolense*. Parasite Immunol.

[B13] Hill EW, O'Gorman GM, Agaba M, Gibson JP, Hanotte O, Kemp SJ, Naessens J, Coussens PM, MacHugh DE (2005). Understanding bovine trypanosomiasis and trypanotolerance: the promise of functional genomics. Vet Immunol Immunopathol.

[B14] O'Gorman GM, Park SD, Hill EW, Meade KG, Mitchell LC, Agaba M, Gibson JP, Hanotte O, Naessens J, Kemp SJ (2006). Cytokine mRNA profiling of peripheral blood mononuclear cells from trypanotolerant and trypanosusceptible cattle infected with *Trypanosoma congolense*. Physiol Genomics.

[B15] Barrett T, Troup DB, Wilhite SE, Ledoux P, Rudnev D, Evangelista C, Kim IF, Soboleva A, Tomashevsky M, Edgar R (2007). NCBI GEO: mining tens of millions of expression profiles – database and tools update. Nucleic Acids Res.

[B16] Lang T, Mansell A (2007). The negative regulation of Toll-like receptor and associated pathways. Immunol Cell Biol.

[B17] Dey R, Khan S, Pahari S, Srivastava N, Jadhav M, Saha B (2007). Functional paradox in host-pathogen interaction dictates the fate of parasites. Future Microbiol.

[B18] Hanotte O, Ronin Y, Agaba M, Nilsson P, Gelhaus A, Horstmann R, Sugimoto Y, Kemp S, Gibson J, Korol A (2003). Mapping of quantitative trait loci controlling trypanotolerance in a cross of tolerant West African N'Dama and susceptible East African Boran cattle. Proc Natl Acad Sci USA.

[B19] Wang H, Frelin L, Pevsner J (1997). Human syntaxin 7: a Pep12p/Vps6p homologue implicated in vesicle trafficking to lysosomes. Gene.

[B20] Wong SH, Xu Y, Zhang T, Hong W (1998). Syntaxin 7, a novel syntaxin member associated with the early endosomal compartment. J Biol Chem.

[B21] Castle A, Castle D (2005). Ubiquitously expressed secretory carrier membrane proteins (SCAMPs) 1–4 mark different pathways and exhibit limited constitutive trafficking to and from the cell surface. J Cell Sci.

[B22] Fernandez-Chacon R, Achiriloaie M, Janz R, Albanesi JP, Sudhof TC (2000). SCAMP1 function in endocytosis. J Biol Chem.

[B23] Kouranti I, Sachse M, Arouche N, Goud B, Echard A (2006). Rab35 regulates an endocytic recycling pathway essential for the terminal steps of cytokinesis. Curr Biol.

[B24] Reyes SL, Leon BF, Rozas VM, Gonzalez JP, Naves PR (2006). BAFF: A regulatory cytokine of B lymphocytes involved in autoimmunity and lymphoid cancer. Rev Med Chil.

[B25] Woodland RT, Schmidt MR, Thompson CB (2006). BLyS and B cell homeostasis. Semin Immunol.

[B26] Fearon DT, Carroll MC (2000). Regulation of B lymphocyte responses to foreign and self-antigens by the CD19/CD21 complex. Annu Rev Immunol.

[B27] Smith KG, Fearon DT (2000). Receptor modulators of B-cell receptor signalling – CD19/CD22. Curr Top Microbiol Immunol.

[B28] Devadas S, Das J, Liu C, Zhang L, Roberts AI, Pan Z, Moore PA, Das G, Shi Y (2006). Granzyme B is critical for T cell receptor-induced cell death of type 2 helper T cells. Immunity.

[B29] Wood P (2006). Understanding immunology.

[B30] Pende D, Parolini S, Pessino A, Sivori S, Augugliaro R, Morelli L, Marcenaro E, Accame L, Malaspina A, Biassoni R (1999). Identification and molecular characterization of NKp30, a novel triggering receptor involved in natural cytotoxicity mediated by human natural killer cells. J Exp Med.

[B31] Moretta A, Bottino C, Vitale M, Pende D, Cantoni C, Mingari MC, Biassoni R, Moretta L (2001). Activating receptors and coreceptors involved in human natural killer cell-mediated cytolysis. Annu Rev Immunol.

[B32] Delahaye NF, Barbier M, Fumoux F, Rihet P (2007). Association analyses of *NCR3 *polymorphisms with *P. falciparum *mild malaria. Microbes Infect.

[B33] Hirst M, Haliday E, Nakamura J, Lou L (1994). Human GMP synthetase. Protein purification, cloning, and functional expression of cDNA. J Biol Chem.

[B34] Diener K, Wang XS, Chen C, Meyer CF, Keesler G, Zukowski M, Tan TH, Yao Z (1997). Activation of the c-Jun N-terminal kinase pathway by a novel protein kinase related to human germinal center kinase. Proc Natl Acad Sci USA.

[B35] Katz P, Whalen G, Kehrl JH (1994). Differential expression of a novel protein kinase in human B lymphocytes. Preferential localization in the germinal center. J Biol Chem.

[B36] Ramjaun AR, Angers A, Legendre-Guillemin V, Tong XK, McPherson PS (2001). Endophilin regulates JNK activation through its interaction with the germinal center kinase-like kinase. J Biol Chem.

[B37] Donovan A, Lima CA, Pinkus JL, Pinkus GS, Zon LI, Robine S, Andrews NC (2005). The iron exporter ferroportin/Slc40a1 is essential for iron homeostasis. Cell Metab.

[B38] Dunn LL, Rahmanto YS, Richardson DR (2007). Iron uptake and metabolism in the new millennium. Trends Cell Biol.

[B39] Jacobs HT, Turnbull DM (2005). Nuclear genes and mitochondrial translation: a new class of genetic disease. Trends Genet.

[B40] Taanman JW (1999). The mitochondrial genome: structure, transcription, translation and replication. Biochim Biophys Acta.

[B41] Cartharius K, Frech K, Grote K, Klocke B, Haltmeier M, Klingenhoff A, Frisch M, Bayerlein M, Werner T (2005). MatInspector and beyond: promoter analysis based on transcription factor binding sites. Bioinformatics.

[B42] Quandt K, Frech K, Karas H, Wingender E, Werner T (1995). MatInd and MatInspector: new fast and versatile tools for detection of consensus matches in nucleotide sequence data. Nucleic Acids Res.

[B43] Geigy R, Kauffmann M (1973). Sleeping sickness survey in the Serengeti area (Tanzania) 1971. I. Examination of large mammals for trypanosomes. Acta Trop.

[B44] Nantulya VM, Musoke AJ, Rurangirwa FR, Moloo SK (1984). Resistance of cattle to tsetse-transmitted challenge with *Trypanosoma brucei *or *Trypanosoma congolense *after spontaneous recovery from syringe-passaged infections. Infect Immun.

[B45] Akol GW, Murray M (1982). Early events following challenge of cattle with tsetse infected with *Trypanosoma congolense *: development of the local skin reaction. Vet Rec.

[B46] Dwinger RH, Murray M, Moloo SK (1987). Potential value of localized skin reactions (chancres) induced by *Trypanosoma congolense *transmitted by *Glossina morsitans *centralis for the analysis of metacyclic trypanosome populations. Parasite Immunol.

[B47] Ulmer AJ, Scholz W, Ernst M, Brandt E, Flad HD (1984). Isolation and subfractionation of human peripheral blood mononuclear cells (PBMC) by density gradient centrifugation on Percoll. Immunobiology.

[B48] Reimers M, Carey VJ (2006). Bioconductor: an open source framework for bioinformatics and computational biology. Methods Enzymol.

[B49] Smyth GK (2004). Linear models and empirical bayes methods for assessing differential expression in microarray experiments. Stat Appl Genet Mol Biol.

[B50] Irizarry RA, Hobbs B, Collin F, Beazer-Barclay YD, Antonellis KJ, Scherf U, Speed TP (2003). Exploration, normalization, and summaries of high density oligonucleotide array probe level data. Biostatistics.

[B51] Yang YH, Dudoit S, Luu P, Lin DM, Peng V, Ngai J, Speed TP (2002). Normalization for cDNA microarray data: a robust composite method addressing single and multiple slide systematic variation. Nucleic Acids Res.

[B52] Benjamini Y, Hochberg Y (1995). Controlling the false discovery rate – a practical and powerful approach to multiple testing. J R Stat Soc Series B Stat Methodol.

[B53] Falcon S, Gentleman R (2007). Using GOstats to test gene lists for GO term association. Bioinformatics.

[B54] Vandesompele J, De Preter K, Pattyn F, Poppe B, Van Roy N, De Paepe A, Speleman F (2002). Accurate normalization of real-time quantitative RT-PCR data by geometric averaging of multiple internal control genes. Genome Biol.

[B55] Livak KJ, Schmittgen TD (2001). Analysis of relative gene expression data using real-time quantitative PCR and the 2(-Delta Delta C(T)) Method. Methods.

